# The Quality Monitoring of Cistanches Herba (*Cistanche deserticola* Ma): A Value Chain Perspective

**DOI:** 10.3389/fphar.2021.782962

**Published:** 2021-11-05

**Authors:** Linlin Jiang, Baochang Zhou, Xiaoqin Wang, Yaqiong Bi, Wenfang Guo, Jianhua Wang, Ruyu Yao, Minhui Li

**Affiliations:** ^1^ Department of Pharmacy, Inner Mongolia Medical University, Hohhot, China; ^2^ Inner Mongolia Hospital of Institute of Traditional Chinese Medicine, Hohhot, China; ^3^ Institute of Medicinal Plant Development, Chinese Academy of Medical Sciences and Peking Union Medical College, Beijing, China; ^4^ Inner Mongolia Key Laboratory of Characteristic Geoherbs Resources Protection and Utilization, Baotou, China; ^5^ Baotou Medical College, Baotou, China; ^6^ Inner Mongolia Hospital of Traditional Chinese Medicine, Hohhot, China

**Keywords:** cistanches herba, quality control, value chain, chemical analysis, specifications and grades, DNA barcoding

## Abstract

Cistanche deserticola Ma was used as a medicine food homology, which was mainly produced in the Alxa region of northwest China. In recent years, it has been widely used in various food items. The increasing demand for Cistanches Herba has led to problems such as overexploitation and quality deterioration. The quality and safety of herbal medicines are critical and have been shown to be affected by the value chain (VC). Using the VC framework, the study is embedded in a larger study aiming to investigate the effects of different VCs types on the quality and stakeholders of Cistanches Herba. In this study, 90 Cistanches Herba samples were collected during fieldwork. An additional 40 samples were obtained from the herbal markets and medicine purchasing stations. Semi-structured interviews and key informant interviews were performed to collect data on stakeholders in major production areas. These samples were analyzed using high performance liquid chromatography (HPLC) coupled with the *k*-means clustering method; a targeted quality assessment strategy based on chemical analysis was adopted to understand the quality of Cistanches Herba. Based on market research, the collected samples were divided into different grades through *k*-means clustering analysis. Moreover, quality differences of Cistanches Herba in Alxa region were explored through DNA barcoding and chemical analysis. Accordingly, 10 different types of VCs were determined in the production of Cistanches Herba. The results show that there is a close relationship between the quality of Cistanches Herba and stakeholder benefits. Vertical integration at different levels was found for independent farmer-based VCs, horizontal collaboration was found in the cooperative-based VCs. The vertical coordination has led to a more consistent traceability system and strict regulation of supply chains. At the same time, the Cistanches Herba were divided into three grades. Through DNA barcoding and chemical analysis, we found that the quality differences between Cistanches Herba in the Alxa area were not significant. It was found that geographical suitability and vertical integration could impact the quality and sustainable production of Cistanches Herba. At the same time, the well-developed VCs can provide products with reliable quality, and ensure adequate financial revenue for relevant stakeholders.

## 1 Introduction

*Cistanche deserticola* Ma (commonly known as *Rou Cong Rong* in Chinese or Cistanches Herba) is a parasitic plant. The dried fleshy stem of *C. deserticola* is a precious traditional Chinese medicine (TCM) that has been used for centuries (Fu et al., 2018). Several pharmacological studies have shown that Cistanches Herba displays a range of functions, including antioxidative, anti-nociceptive, anti-inflammatory, immunity, neuroprotective, and hepatoprotective functions (Li Z. et al., 2016; You et al., 2016). Cistanches Herba was used as a homology of medicine and food in 2018, which indicates that in addition to curing specific diseases, it can also be consumed daily for health care needs (Li et al., 2021). In recent years, it has been widely used in various food items, tea, drinks, and beverages. *Rou Cong Rong* tea is becoming increasingly popular as a health food supplement in Asian countries, such as China and Japan (Li et al., 2018; Hou et al., 2020; Lei et al., 2020). Cistanches Herba is known as desert ginseng due to its nutritional value (Wang et al., 2012). Consequently, Cistanches Herba is popularly used in TCM and health care practices.

With increasing interest in the clinical and health care functions of Cistanches Herba, more research is focused on its active ingredients. Phenylethanoid glycosides comprise a significant class of compounds that exhibit a wide range of pharmacological effects (Li Z. et al., 2016). Echinacoside and acteoside are the main active ingredients of Cistanches Herba. These are described as “marker components” in the Chinese Pharmacopeia 2020 edition (ChP., 2020) (Wang X. Y. et al., 2017). Galactitol is the main laxative constituent of Cistanches Herba. At the same time, it also performs a variety of biological activities, such as anti-aging, and regulation of immune function. Galactitol exhibits low toxicity and minimal side effects and has broad prospects in clinical applications and health food development (Gao et al., 2015). Thus, determination of galactitol confers a certain significance to the development and utilization of Cistanches Herba.

The demand for Cistanches Herba in the global market has grown rapidly, whereby the annual demand for Cistanches Herba is bordering on approximately 4,000 tons (Lu et al., 2019). However, the number of its hosts has been declining, as they are being cut down to be used as firewood by herdsmen. The populations of high-value plants that grow in the wild have rapidly declined due to the high market demand for medicinal plants, resulting in their scarcity in the market (Ndou et al., 2019). As a result, with the increase in demand year by year, the wild *C. deserticola* populations have been seriously affected, and the wild resources have been gradually exhausted. It has been listed as a “second grade” nationally protected plant in China, the International Union for Conservation of Nature Red List of Threatened Species and Convention on International Trade in Endangered Species of Wild Fauna and Flora (Appendix II) (Fan et al., 2020), resulting in restrictions being imposed on international trade. This has led to long-term stagnation of *C. deserticola*-related industries. Thus, artificial cultivation of *C. deserticola* is important. Li et al. (1989) have studied the artificial cultivation of *C. deserticola* since the 1980s. In the 1990s, researchers systematically elucidated the parasitic mechanism of *C. deserticola*, explored the key technologies associated with the artificial cultivation of parasitic plants, and established a sustainable *C. deserticola* cultivation system that produces high yields. At present, the artificial inoculation technology for *C. deserticola* cultivation is becoming increasingly advanced, and the cultivated area is expanding on a yearly basis. Therefore, artificial cultivation of *C. deserticola* may be considered to resolve issues arising from a scarcity of medicinal and protected wild resources (Bi et al., 2020). Moreover, the development of a series of health products based on Cistanches Herba has resulted in a substantial expansion in the international influence of Cistanches Herba.

*C. deserticola* has been cultivated in several plantations to meet the increasing demands of international and domestic herbal medicine markets (Hou et al., 2020). The quality of Cistanches Herba is affected by growing conditions, harvest seasons, and processing methods (Li et al., 2019). Due to natural conditions, ecology, and cultivation techniques, the Cistanches Herba in Alxa have larger and fleshier stems, are richer in pectin and tannin, and are of good quality. Therefore, Alxa is considered to be the ideal location for growing and producing top quality Cistanches Herba (*Daodi* herbs) (Li et al., 2019). *Daodi* herbs have self-adapting characteristics to adapt to their environments over long periods of time. Thus, they reportedly display advanced properties, such as superior active ingredients and better quality (Yin et al., 2019). Plants vary greatly in chemical composition; thus, any quality assessment must take the length, diversity, and uncertainty of value chains (VCs), from which these plants originate, into account (Heinrich et al., 2019). Recent studies have indicated that the VCs of medicinal plants have a significant impact on the quality and safety of herbal medicinal products, such as *Curcuma longa* L., *Ginkgo biloba* L., and *Lycium barbarum* L. (Booker et al., 2014; Booker et al., 2016; Yao et al., 2018). Improvements in medicinal quality and safety add value to the final product. Hence, maintaining supply of prime materials via VCs is considered vital for meeting the increasing demand for Cistanches Herba.

Starting from cultivation to processing and distribution, a medicinal plant product must pass through several levels of stakeholders before it reaches the final consumer (Székács et al., 2018). Besides production, VC research also focuses on other activities related to the supply chain, including distribution and marketing. VCs define the activities involved in different modes of production, while emphasizing the relationship between primary producers and other stakeholders in different production systems and their socio-economic impact (Bi et al., 2020). Although VC analyses have been widely used in various products, only a few studies have investigated the VCs of medicinal plants and their derivatives. It was not until 2012 that people became increasingly aware of the broader role of VCs associated with medicinal plants in global markets (Booker et al., 2012; Booker et al., 2015).

Medicinal plants and their derived products appear to have varied VCs. Within a VC, there may be different stakeholders who contribute to different stages of supply. Understanding the relationship between production and supply helps identify the pressure points in different VCs. By comparing different VCs horizontally and vertically, a better understanding of the differences in the quality and price of herbal medicines in different markets may be acquired (Yao et al., 2018; Bi et al., 2020). In a short chain, such as one where the product is grown and used locally as an herbal medicine, the quality and safety of the product is relatively easy to control and manage. In contrast, using a TCM or food supplement in the domestic market or as an export product involves greater risks and challenges (Booker et al., 2015; Heinrich et al., 2019). It is worth noting that the maximum allowable levels of contaminants, authenticity, and purity are usually controlled at a very late stage. Only careful management along VCs allows improvement in the quality and safety of cultivated medicinal plants.

Alxa, being the main production area, accounts for more than 90% of the total output of Inner Mongolia and more than 70% of China’s total output (Siqin et al., 2019). It is mainly cultivated via wild cultivation mode, without using chemical fertilizers or pesticides, and has great economic and ecological value. Due to its high medicinal value and nutritional effects, adulteration of Cistanches Herba often occurs under market conditions, which limits the safety and sustainable development of the products. There is a lack of information on the quality risks arising from these systems and risk management in production systems and VCs. In the current study, we aimed to investigate the quality differences in Cistanches Herba from the perspective of VCs. We investigated its production and circulation in the daodi area (Alxa) and analyzed specific issues affecting quality and yield. Differences in multiple stakeholders, capital flows, and information were clarified according to VCs. In addition, market research enabled us to develop a Cistanches Herba grading system; a targeted quality assessment strategy based on chemical analysis was adopted to understand the quality of Cistanches Herba, which provided a basis for ensuring and improving quality as well as the economic benefits of production and consumption.

## 2 Materials and Methods

### 2.1 Fieldwork

Based on documentary research and on-site visits, starting August 2016, fieldwork was conducted during the cultivation and harvesting periods in Inner Mongolia, Gansu, Ningxia, and Xinjiang, covering the main production areas as well as several trading centers associated with Cistanches Herba. To maintain variability within an acceptable range, samples for content determination were collected only during the spring. We collected 90 samples, including 18 from Alxa Left Banner (105°42′E/38°50′N), 50 from Alxa Right Banner (101°68′E/39°20′N), and 22 from Ejina Banner (100°88′E/41°90′N). Moreover, we visited three national herbal markets, namely the Yulin Chinese herbal medicine market, Bozhou Chinese herbal medicine market, and An’guo Chinese herbal medicine market. The standard for grading Cistanches Herba was established by measuring the morphological characteristics of the samples. Participatory observations and semi-structured interviews were conducted with different stakeholders. Farmers, wholesalers, and retailers from core cultivation areas or trading centers of the visited areas were randomly selected to collect general information on VCs, current market conditions, and grade classification of Cistanches Herba.

### 2.2 Plant Material

90 Cistanches Herba samples were collected in Alxa during fieldwork. An additional 40 samples were offered either by the institutions we visited and bought in the above mentioned herbal markets and seven medicine purchasing stations in Inner Mongolia, Gansu, Ningxia, and Xinjiang. Whole Cistanches Herba plants were identified by Prof. Wang of Baotou Medical College. The voucher specimens were preserved at the Baotou Medical College, University of Inner Mongolia.

### 2.3 VCs Analysis

Analysis of VCs was carried out via the following steps: 1) identification of the main links in the production of Cistanches Herba; 2) identification of stakeholders in the production process and adding them to the corresponding process; 3) calculation of labor and non-labor costs of each transaction link based on collected data and information; and 4) building of a framework for the VCs to analyze VCs in relation to financial performance, production behavior and quality (Yao et al., 2018). In addition, the strengths and weaknesses of the different VCs were analyzed.

### 2.4 Reagents and Chemicals

A HPLC system was purchased from Thermo Fisher Scientific (Ultimate 3000, United States). A pulverizer (ST-08, 1800 W, China), laboratory water purifier (AMFI-5-P, Yiyang Enterprise, China), electronic analytical balance (AR153O, Mettler Toledo), Retsch-MIVMOO ball mill (model MM400, Retsch, Germany) and desk centrifuge (TGL-16G, Shanghai Anting) were also used. Methanol and acetonitrile were of HPLC grade, (Tianjin Kemiou Chemical Reagent Co., Ltd.) while all other reagents were analytical grade. The reference compounds echinacoside, acteoside, and galactitol were purchased from Chengdu Pufei De Biotech Co., Ltd. (Chengdu, China).

### 2.5 Quantitative Analyses of Main Chemical Indexes in Cistanches Herba

Echinacoside and acteoside were detected under the following conditions: analysis was performed using a C18 column at a flow rate of 1.0 ml/min; the mobile phase was 35% methanol-0.1% acetic acid; the column temperature was set at 30°C; and the DAD detector wavelength was 334 nm. Conditions for the determination of galactitol were as follows: sample analysis was carried out on a Prevail Carbohydrate ES polymer gel column at a flow rate of 0.7 ml/min, the mobile phase was acetonitrile-water (77: 23), and the column temperature was set to 25°C. An Alltech ELSD 2000ES evaporation light scattering detector (Alltech Technology Ltd.) was used, the drift tube temperature was set to 40°C, and the gas pressure was set to 240 kPa.

### 2.6 Molecular Identification of Cistanches Herba

#### 2.6.1 DNA Extraction, PCR Amplification and Sequencing

Genomic DNA was extracted from silica gel-dried medicinal materials. Approximately 0.03 g of the molecular material was ground for 45 s at a frequency of 3,000 times/s in a Retsch-MIVMOO ball mill. DNA extraction was performed using an optimized cetyl trimethylammonium bromide method. Then, *rbc*L, *mat*K, *trn*H-*psb*A, and ITS regions were amplified by polymerase chain reaction (PCR) with primers. A 25 μL PCR amplification was performed. Purified PCR products were sequenced in the forward direction using the primers used for amplification on an ABI3730XL sequencer.

#### 2.6.2 Phylogenetic Analysis

In this study, *Kopsiopsis hookeri* (Walp.) Govaerts, *Xylanche himalaica* (Hook. f. & Thomson) Beck, *Cistanche tinctoria* (Forssk.) Beck, and *Cistanche salsa* (C. A. Mey.) Beck were selected as the outgroup for *rbc*L, *mat*K, *trn*H-*psb*A, and ITS, respectively. A phylogenetic tree of four sequences of Cistanches Herba was constructed using the neighbor-joining (NJ) method with MEGA 6.0 software. The support rate of each branch was tested via 1,000 repetitive bootstrap tests, and 50% support was used as the threshold for successful identification.

### 2.7 Clustering Analysis

The *k*-means algorithm is capable of effectively analyzing large-scale data and is easy to understand. Automatic grouping was carried out according to the degree of natural affinity, which makes the structural characteristics of individuals within the group very similar, and the characteristics of the individuals between the groups different (Wang T. et al., 2017). *k*-means clustering was performed using SPSS and Rstudio to analyze market grades and the relationship between the contents of the active ingredients of the field sample and its place of origin.

## 3 Results and Discussion

### 3.1 Industrial Structure and VCs

**FIGURE 1:**
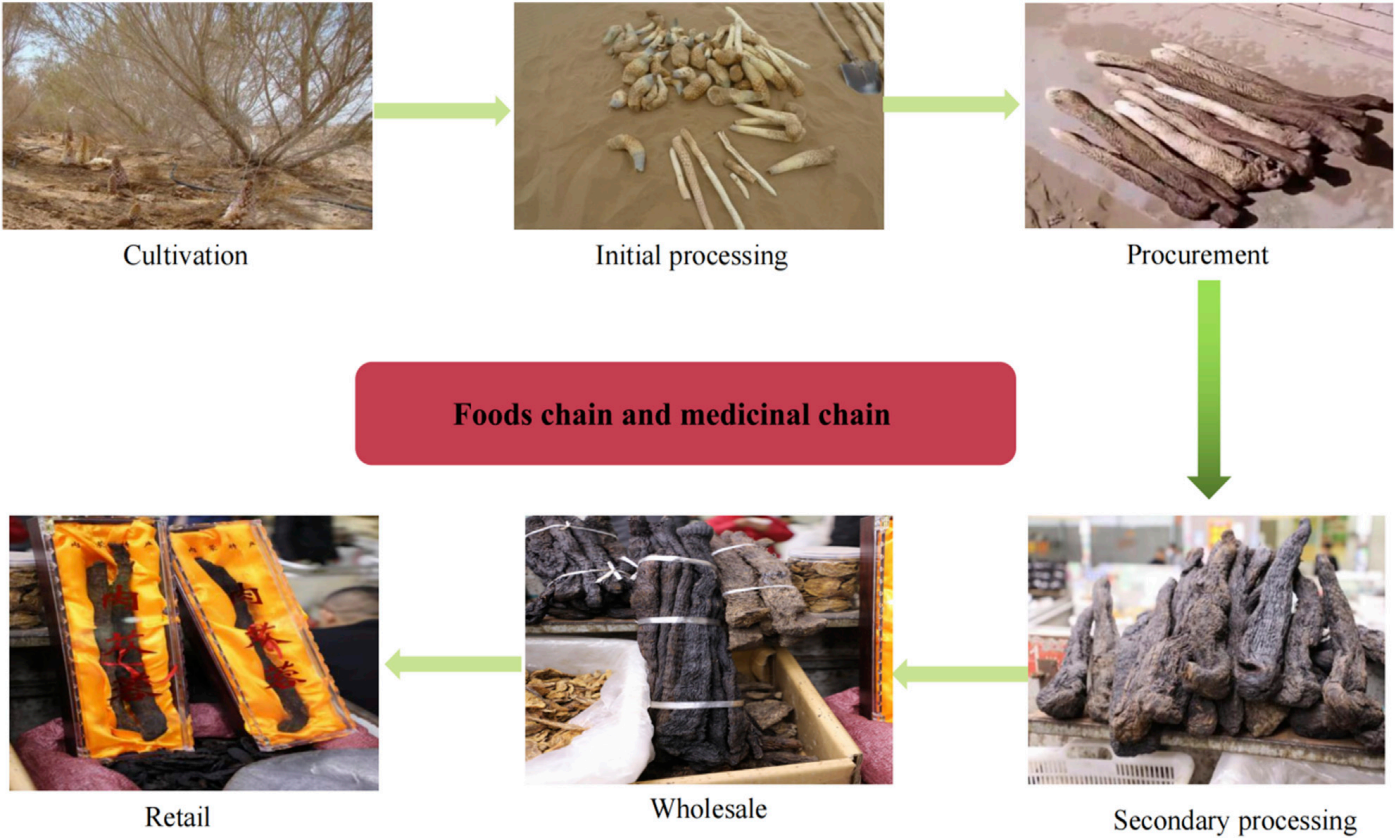
Cistanches Herba goes through six production stages in food and medicinal chain.

Cistanches Herba goes through six stages before it reaches the consumer as follows: 1) cultivation, including activities, such as plowing, inoculation, pruning, rodent control, and harvesting; 2) initial processing, including surface treatment, removing impurities, as well as drying; 3) procurement, which involves trading of unprocessed material produced in step 2; 4) secondary processing, including commercial grading, deep-processing and packaging; 5) wholesale, mainly to nonlocal markets; and 6) retail (Figure 1).

**FIGURE 2:**
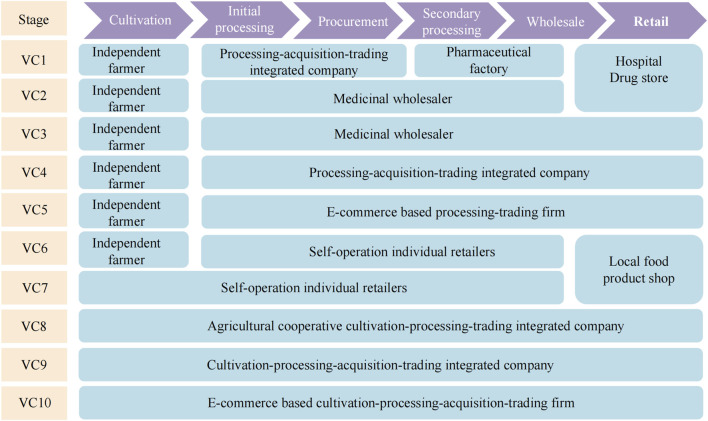
Cistanches Herba production involves 10 mature VCs.

On account of its long production and supply lines, Cistanches Herba production involves 10 mature VCs, which may be distinguished by the various composite patterns of stakeholders. Different stakeholders play a role in each of these steps, resulting in various forms of VCs. The 10 primary VCs identified as being associated with Cistanches Herba as well as the stakeholders involved are shown in Figure 2.

VCs 1–6 are based on independent farmers with small Cistanches Herba fields offering harvested products to medicinal wholesalers, local individual retailers or other cultivation-processing-trading integrated companies. VC 7 is run by local individual retailers. Harvested products are often sold as local food products. VC 8 initiates with farmers involved in agricultural cooperatives (with relatively large fields). VCs 9 and 10 are characterized by cultivation-processing-acquisition-trading firms.

Independent farmers with relatively small fields are placed in VCs 1–6. These tend to be traditional, small-scale economies. In these VCs, independent farmers grow and harvest Cistanches Herba. Farmers near local individual retailers, medicinal wholesalers, or processing companies sell their products directly, without the involvement of middlemen, to increase profits earned from the cultivation of this plant. In VC 1, unprocessed Cistanches Herba is sold to processing firms; Cistanches Herba is processed into raw materials and sold to pharmaceutical factories, and then the products are sold to consumers through pharmacies and the like. Due to the particularities of the growing environment and strict storage requirements, many individual retailers, such as those in VCs 2, 3 and 6, are able to procure unprocessed Cistanches Herba directly from farmers, and reduce intermediate links, thus improving the quality. It is then further processed into herbal medicines or functional food and sold to consumers, hospitals, or pharmacies. At the same time, some are dried and processed as agricultural by-products for sale. However, farmers directly sell their products without processing, and the product quality is likely to be not stable due to insufficient drying during the transportation process. Additionally, the development of e-commerce makes it possible for a processing firm to expand downstream. VC 5, the trading firm plays a role that extends up to the retailer.

VC 7 is a one-stop VC conducted by self-operating individual retailers who hire local farmers or carry out their own production and processing. They usually sell their products through their own offline and online stores, which play an important part in a sale. These products are usually bought directly by consumers in the form of local agricultural products and functional food products.

VC 8 begins with agricultural cooperative-based VCs. Related governmental departments provide farmers with beneficial policies to plant Cistanches Herba. Thus, enterprises are encouraged to develop agricultural cooperatives. These farmers receive more technical support and equipment from cooperatives than independent farmers.

VC 9 and VC 10 are regulated by companies that control cultivation, processing and trading, and are based in large production areas. They further process the products into functional food products, which are then directly sold to retailers or consumers, mainly through chain stores or online stores. Moreover, some products are sold to foreign wholesalers.

### 3.2 Trading along with VCs of Cistanches Herba

The value of a product is created by the production activities of the stakeholders and is generated during the transaction process. In different VCs, labor input and non-labor input have different effects on value (Yao et al., 2018).

Alxa has a vast land area and rich natural resources, which lays the foundation for its ecological construction and the development of the deserticulture industry. Herdsmen mostly contract pastures, where there are large areas of natural *Haloxylon ammodendron* (C. A. Mey.) Bunge ex Fenzl, as well as a large space suitable for planting *H. ammodendron*. Farmers usually plant *H. ammodendron* in their own fields or grasslands. After *H. ammodendron* survives, it is grafted onto *C. deserticola*, and finally the industrial model is based on selling *C. deserticola* raw materials. Therefore, the cultivation of Cistanches Herba is one of the income sources of herdsmen (Zhao and Zhang, 2014). However, the cultivation of Cistanches Herba is a long-term investment owing to the long planting cycle, large capital demand, and slow harvest.

During the planting period of *H. ammodendron*, a large amount of water is needed for irrigation. Owing to the serious shortage of groundwater in Alxa, the land can only be irrigated manually. At the same time, since *H. ammodendron* forests are more scattered and the area is larger, it is necessary to have the protection of fencing because the new young *H. ammodendron* seedlings planted every year cannot survive the grazing of animals. In the survey, many farmers and herdsmen generally reported serious issues with rats in *H. ammodendron* forests. Therefore, in the early stage of planting, the cost of watering, fencing, rodent control, machinery, and human labor in *H. ammodendron* forests are higher, resulting in higher costs for Cistanches Herba production. However, the wild cultivation model can save a portion of the non-labor costs of chemical fertilizers and pesticides while ensuring high quality. At the same time, the Alxa government pays a certain amount of compensation to the Cistanches Herba cultivators. However, Cistanches Herba cultivated by independent farmers has lower survival rates and yields than that cultivated by cooperative companies, which mainly leads to lower income for independent farmers.

In addition, some stakeholders, such as agricultural cooperatives and integrated cultivation-processing-trading companies, have well-established facilities that help reduce labor costs and lower inputs, thus guaranteeing high returns. By contrast, VCs 8–10 involve large-scale agricultural operations that are increasingly popular, including agricultural cooperatives and plantation companies. In addition to providing a traditional medicinal market, further processing into functional food adds to its benefits and efficiency.

Processing and wholesale are also labor-intensive stages (20–25 Chinese yuan (CNY)/kg). Before they move on to the second processing stage, processing companies spend a large amount of funds on production and testing equipment, production workshops and storage rooms. Storage of Cistanches Herba is an important factor affecting quality and price, and therefore a large amount of capital and labor is invested in storage. In addition, these factories must perform quality testing for each batch of TCM. At the same time, part of the product is processed into the food market. In addition, a large part of the input was made in the management of the factory.

Retail is the final stage of the VC. Here, the resources flow to high-quality hospitals, traditional herbal medicine markets, agricultural markets, and online markets. The trends seen in prices indicate that these are related to the dynamic balance between demand and supply. It is thus believed that as the demand for Cistanches Herba products grows, the price (and the economic benefits to farmers) will continue to rise in the short term. However, over the long term, progressively more Cistanches Herba will be harvested, causing prices (and farmers’ financial gains) to decline.

The retail price of Cistanches Herba is generally higher than its wholesale price. The price of Cistanches Herba appreciates with grade. The market price of an entire plant is higher than that of its slices. An increase of 10 cm in the length of Cistanches Herba is equivalent to an increase of 100 CNY. In addition, slicing position and processing method causes a difference in the product phase, exerting a significant impact on the price. The size of Cistanches Herba shrinks sharply after drying in the shade or in the sun. The surface of the slices are obviously wrinkled, the color deeper, and the price is the lowest. After harvesting at the site of production, it is cleaned, peeled, and dried after being sliced. If the surface of the slice is smooth, then the price is intermediate. In pharmacies and hospitals, Cistanches Herba is further processed into decoction pieces or formula granules, and the retail price becomes generally twice the wholesale price.

### 3.3 Quality Evaluation of Cistanches Herba Along VCs

#### 3.3.1 Grades of Cistanches Herba

**FIGURE 3:**
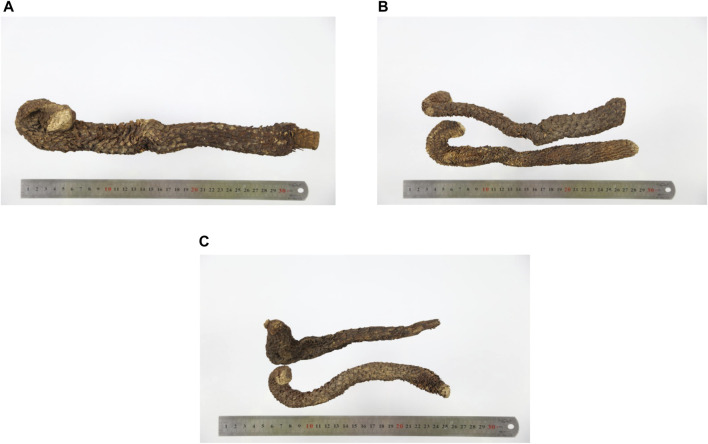
Different grades of Cistanches Herba (**(A)**: first class, **(B)**: second class, **(C)**: third grade).

In Alxa areas, the wild cultivation mode, without the use of chemical fertilizers or pesticides, helps sustainable development. Alxa has been considered as the *daodi* area of Cistanches Herba and has a reputation as “the hometown of Cistanches Herba in the world” (Siqin et al., 2019). Cultivation according to suitable ecology is a factor that guarantees the yield and quality of Cistanches Herba. However, the current planting of Cistanche Herba in Alxa has not been standardized. Therefore, the disordered classification of Cistanches Herba in the market leads to price differences. Currently, the size of obtainable medicinal materials is generally used as the main basis for grading (Li et al., 2019). We conducted an in-depth study of all samples collected. Based on requirements, the morphology, surface characteristics and size, and parameters, such as diameter and length, of Cistanches Herba were analyzed (Yan et al., 2019). More precisely, Cistanches Herba was divided into three grades (Figure 3). These results may be used to determine the specifications and grades of Cistanches Herba (Table 1). At the same time, the market survey found that the price of first-grade products was more than 200 CNY/kg; second-grade products cost about 180 CNY/kg; and third-grade products cost lower than 150 CNY/kg. By analyzing the quality differences between Cistanches Herba stocks in the market, a more reasonable standard for grading Cistanches Herba was established to guide a more standard production mode.

**TABLE 1:** The specification and grade of Cistanches Herba.

Name	Grade	Shape	Surface character	Texture	Cross section	Taste	Size
Cistanches Herba	First grade	Flat cylindrical, slightly curved	The surface is brown or gray-brown, by most imbricate arrangement fleshy scaled leaf. Typically, the scales tip has been broken leaf	Hard, slightly flexible, not easy to break	The cross section is brown, with light brown punctate vascular bundles, arranged into undulate rings	Sweet, slightly bitter	length is greater than 25 cm, and the diameter is greater than 3.5 cm
	Second grade						length is 15–25 cm, and diameter is 2.5–3.5 cm
	Third grade						length is 3–15 cm, and the diameter is greater than 2 cm

#### 3.3.2 Quality Evaluation of Cistanches Herba

*Daodi* herbs has been widely recognized by the Chinese medicinal industry as a symbol of stable, high-quality TCM for a long time (Yin et al., 2019). Along the VCs, different criteria were considered as being indicative of good quality. For farmers, size is often a measure of quality; the bigger the fleshy stem, the better the quality. Retailers believe that habitat and size are important indices of the quality of Cistanches Herba. In addition, doctors believe that better quality would have a more effective clinical effect. At present, these measures can be used to evaluate the quality of Cistanches Herba that have been established in pharmacopeia. The quality of Cistanches Herba has been evaluated using methods, such as source, morphological, and physicochemical identification in the pharmacopeia (ChP., 2020). In addition, some planting companies and pharmaceutical factories impose their own regulations to ensure the quality of their herbal medicines. However, independent farmers are technologically not proactive enough to access them.

Along the VCs, different criteria were considered as being indicative of good quality. Therefore, a quality investigation of Cistanches Herba from different VCs was performed, and the results are shown in Table 2. The quality of Cistanches Herba is related to planting, storage, drying, and processing. In VCs 1–4, farmers directly sell their products without processing, and the product quality is not stable due to insufficient drying during the transportation process. In VCs 5 and 6, Cistanches Herba is usually sold as an agricultural product, making it difficult to trace or supervise effectively. In VCs 8–10, the integrated chain of cultivation, harvesting, and processing may reduce the loss of active ingredients. In addition, deep processing enterprises will have a stricter traceability and quality assurance system for products, thus guaranteeing quality.

**TABLE 2:** Quality of the Cistanches Herba for the different VCs and likelihood of risks being made to its quality during its production.

VC	Traceability	Certify	Control	Heavy metal	Pesticide residue	Likelihood of hazard occurring		
						Cultivation	Processing	Wholesale
1	No	Maybe	Medium	No	No	Improbable	Probable	Probable
2	No	No	Weak	No	No	Improbable	Very probable	Very probable
3	No	No	Weak	No	No	Improbable	Very probable	Very probable
4	No	Maybe	Medium	No	No	Improbable	Probable	Probable
5	No	Maybe	Medium	No	No	Improbable	Probable	Very probable
6	No	No	Weak	No	No	Improbable	Probable	Probable
7	No	No	Weak	No	No	Improbable	Probable	Probable
8	Maybe	Maybe	Strong	No	No	Improbable	Improbable	Improbable
9	Yes	Maybe	Strong	No	No	Improbable	Improbable	Improbable
10	Yes	Maybe	Strong	No	No	Improbable	Improbable	Probable

Generally, the effective ingredient content of a plant is related to its growth period (Li Z. et al., 2016). Therefore, the evaluation of the main chemical components of Cistanche Herba can reflect its quality to a certain extent. Echinacoside and acteoside are the main active ingredients of Cistanches Herba (Li Y. et al., 2016). These are described as marker components in the Chinese Pharmacopeia 2020 edition (Wang X. Y. et al., 2017). Galactitol is the main laxative constituent of Cistanches Herba. Thus, determination of galactitol confers a certain significance to the development and utilization of Cistanches Herba (Gao et al., 2015). Moreover, a one-way analysis of variance of active ingredient content and characteristics (Table 3), indicated that the active ingredient content was correlated with the characteristics of Cistanches Herba. The higher the oiliness of the sample, the higher the content of its chemical components. At the same time, the study found that galactitol content is related to the weight of the sample, which further illustrates the significance of galactitol content determination.

**TABLE 3:** One-way analysis results of variance between appearance of Cistanches Herba and echinacoside, acteoside, galactitol.

Compounds	Oiliness	Weight	Density of scales	Size	Texture
Echinacoside (%)	−1.336	1.152	0.179	0.287	0.644
Acteoside (%)	−2.445*	0.341	3.277*	0.880	1.947
Galactitol (%)	−2.254*	3.577*	0.626	0.464	0.551
The total contents (%)	−2.099*	0.744	1.317	0.444	1.455

*p < 0.05.

**FIGURE 4:**
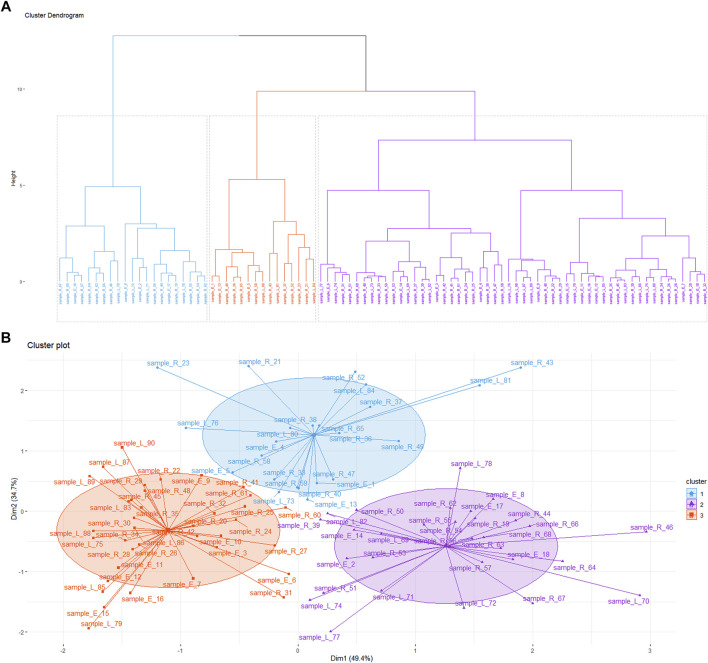
**(A,B)** Results of the constituents of Cistanches Herba based on *k*-means cluster (sample_E: the sample of Ejin Bannerr; sample_R: the sample of Alxa Right Banner; sample_L: the sample of Alxa Left Banner).

We determined the content of all samples collected from the field. The results showed that the content of active ingredients in Alxa was high (Supplementary Table S1), clarifying the high quality of Alxa in the market and the high prices paid. *k*-means cluster analysis was used to analyze 90 samples collected from the field based on three variables: echinacoside, acteoside, and galactitol (Figures 4A,B). These results show that the samples can be divided into three different clusters, but the clusters representing Cistanches Herba from the three regions are relatively disorganized. Analysis of the chemical components of Cistanches Herba from different areas of Alxa revealed that there was no significant correlation between chemical composition and geographical distance. We believe that there is no substantial difference between in the quality of Cistanches Herba from different areas of Alxa.

**FIGURE 5:**
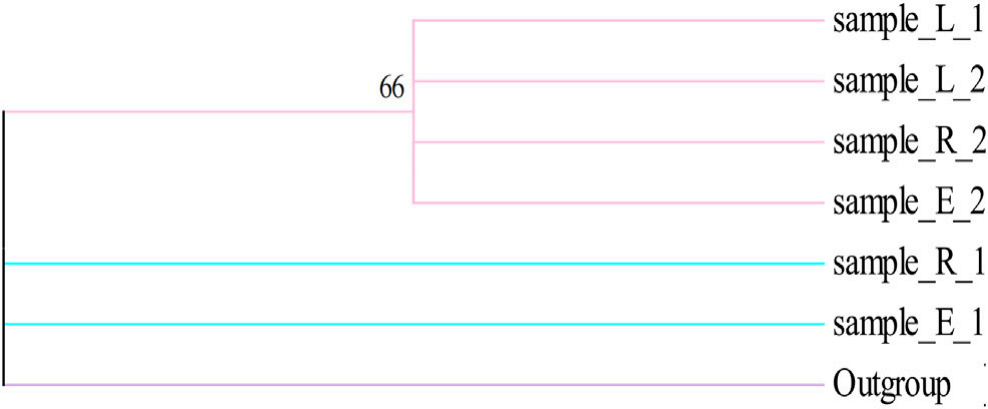
Phylogenetic tree of Cistanches Herba based on the ITS sequences.

**FIGURE 6:**
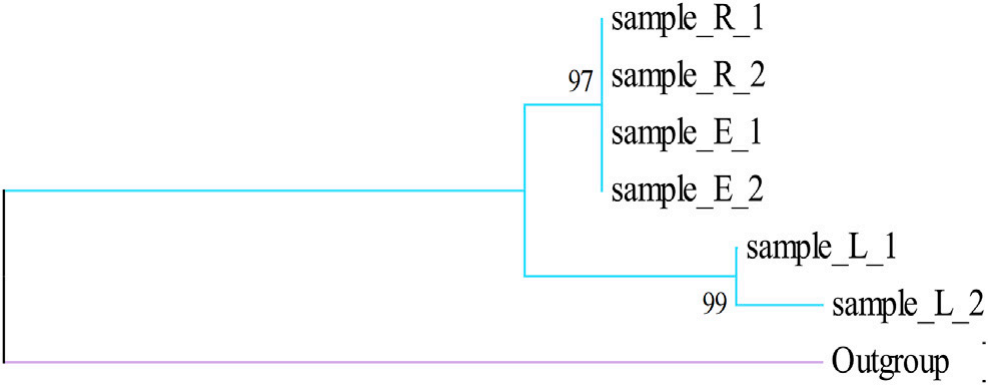
Phylogenetic tree of Cistanches Herba based on the *trn*H-*psb*A sequences.

**FIGURE 7:**
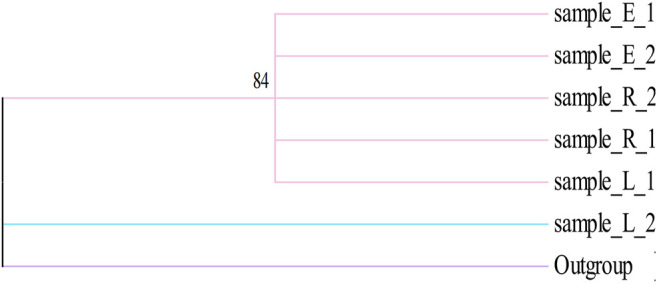
Phylogenetic tree of Cistanches Herba based on the *rbc*L sequences.

**FIGURE 8:**
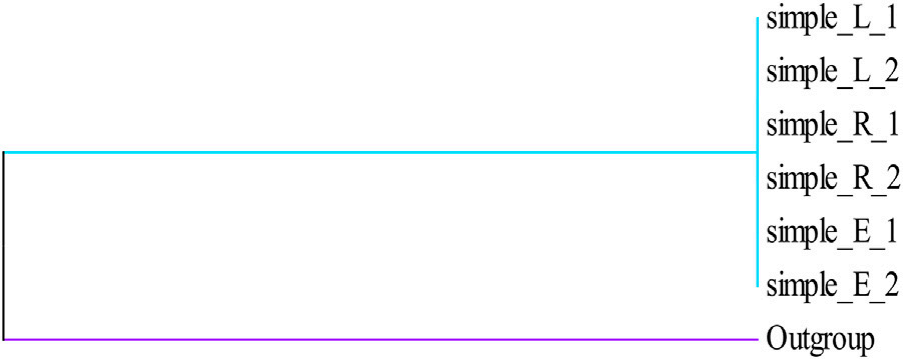
Phylogenetic tree of Cistanches Herba based on the *mat*K sequences.

Herbal products contain numerous ingredients, and phytochemical analyses used in the quality control of marker compounds are accurate but have limited applicability in the identification of biological constituents. The herbal products, sold worldwide as medicines or foods, are perceived as low risk because they are considered natural and thus safe (Ichim, 2019). The growing demand for herbal products due to their health benefits has resulted in a proportionally increase of their accidental contamination or intentional adulteration. To address this problem, DNA barcoding as powerful strategy has recently attracted considerable attention and, along with chemical methods, has started to enter the regulatory systems for quality control (Ichim et al., 2020). DNA barcoding is widely used for molecular identification for the purpose of solving a broad range of issues in taxonomy, molecular phylogenetics and population genetics, as well as for preventing illegal wildlife collection and product authenticity monitoring (Janjua et al., 2017; Raclariu et al., 2018). In this study, we randomly selected a few samples for DNA barcoding identification using four DNA barcode sequences (*rbc*L, *mat*K, *trn*H-*psb*A, and ITS) to identify Cistanches Herba from Alxa habitats. A *trn*H-*psb*A sequence analysis indicated a deletion in Alxa Left Banner at 298–337 bp, compared with Alxa Right Banner and Ejina. It remains to be verified whether this can be used as a specific identification site for Cistanches Herba. Based on the NJ tree established by the sequence, the sequence clustering of the four fragments of the sample cannot be distinguished (Figures 5–8). In addition, the genetic distance analysis results showed that there was no significant correlation between genetic distance and geographical distance (Table 4–7). A previous study of ours found that site-specific PCR may be used to screen site-specific primers for the purpose of identifying *C. deserticola*, *Cynomorium coccineum subsp. songaricum* (Rupr.) J. Léonard, and *Orobanche pycnostachya* Hance based on ITS sequences (Li et al., 2014). However, the identification of Cistanches Herba remains to be further studied because of the existence of other adulterants. By studying the DNA barcoding of Cistanches Herba, we found that the quality differences between Cistanches Herba populations in the Alxa area were not significant. We also quickly and accurately identified Cistanches Herba and its adulterants, which is of vital importance for guaranteeing food and medicine safety and regulating market circulation.

**TABLE 4:** Genetic distance of Cistanches Herba based on ITS sequences.

	Sample_L_1	Sample_L_2	Sample_R_1	Sample_R_2	Sample_E_1	Sample_E_2
Sample_L_1						
Sample_L_2	0.000000					
Sample_R_1	0.002336	0.002336				
Sample_R_2	0.000000	0.000000	0.002336			
Sample_E_1	0.002336	0.002336	0.000000	0.002336		
Sample_E_2	0.000000	0.000000	0.002336	0.000000	0.002336	
Outgroup	0.004683	0.004683	0.002336	0.004683	0.002336	0.004683

(sample_E: the sample of Ejin Bannerr; sample_R: the sample of Alxa Right Banner; sample_L: the sample of Alxa Left Banner).

**TABLE 5:** Genetic distance of Cistanches Herba based on *trn*H-*psb*A sequences.

	Sample_L_1	Sample_L_2	Sample_R_1	Sample_R_2	Sample_E_1	Sample_E_2
Sample_L_1						
Sample_L_2	0.017571					
Sample_R_1	0.000000	0.017571				
Sample_R_2	0.000000	0.017571	0.000000			
Sample_E_1	0.000000	0.017571	0.000000	0.000000		
Sample_E_2	0.000000	0.017571	0.000000	0.000000	0.000000	
Outgroup	0.421739	0.421739	0.421739	0.421739	0.421739	0.421739

**TABLE 6:** Genetic distance of Cistanches Herba based on *rbc*L sequences.

	Sample_L_1	Sample_L_2	Sample_R_1	Sample_R_2	Sample_E_1	Sample_E_2
Sample_L_1						
Sample_L_2	0.019521					
Sample_R_1	0.000000	0.019521				
Sample_R_2	0.000000	0.019521	0.000000			
Sample_E_1	0.000000	0.019521	0.000000	0.000000		
Sample_E_2	0.000000	0.019521	0.000000	0.000000	0.000000	
Outgroup	0.047834	0.064703	0.047834	0.047834	0.047834	0.047834

**TABLE 7:** Genetic distance of Cistanches Herba based on *mat*K sequences.

	Sample_L_1	Sample_L_2	Sample_R_1	Sample_R_2	Sample_E_1	Sample_E_2
Sample_L_1						
Sample_L_2	0.002391					
Sample_R_1	0.001194	0.001194				
Sample_R_2	0.000000	0.002391	0.001194			
Sample_E_1	0.000000	0.002391	0.001194	0.000000		
Sample_E_2	0.000000	0.002391	0.001194	0.000000	0.000000	
Outgroup	1.709952	1.709952	1.709952	1.709952	1.709952	1.709952

In conclusion, the genetic distance and chemical composition of Cistanches Herba were not correlated with geographical location in Alxa. However, this result may be limited by our representative sample and the availability of the sequences. Thus, these results require further verification.

### 3.4 Relationship Between Behavior, Revenue, and Quality

Cistanches Herba was considered both a medicine and food (Li et al., 2021). The main retail channels of Cistanches Herba include hospital pharmacies, drug stores, medicine markets, agricultural by-product, markets, and online shops. Currently, due to its high medicinal value and nutritional function, a trend for developing Cistanches Herba is gradually forming in the form of traditional, food, and deep processing markets. Among these three major markets, due to the yield, geo-herbalism, and clinical function factors, the price of *C. deserticola* from Alxa is higher than that of *C. tinctoria* in the traditional market and *C. deserticola* rarely enters traditional markets. Usually, local people choose good quality Cistanches Herba as gifts from local retail outlets and pay a higher price.

The objectives of all stakeholders in these types of VCs are centered on profitability. However, in the traditional market, independent farmers generally adopt the sales mode of selling raw materials directly, which has low additional value. In the food products market, these mainly enter the market in the form of gifts, which are often higher in quality and price than those sold in the traditional market. In deep-processing-oriented chains, quality and additional value products are improved, thus increasing overall cost. Producers of deep-processed Cistanches Herba typically receive higher revenue from consumers, while stakeholders in traditional markets receive less. Therefore, the behavior and benefits of stakeholders are related to the quality of the product and the target market.

Regarding VC 1, some of the primary processing products are sold as raw materials to pharmaceutical factories by the primary processing companies, which are then further processed into other forms of drugs that bring greater profits. However, selling these products as drugs is often affected by higher regulations.

VCs 2 and 3 are typical examples of traditional markets, where medical wholesalers buy herbs directly from farmers. In these chains, it is difficult for farmers to expand the planting scale, due to the long planting cycle of Cistanches Herba, which requires high levels of early investment in time and labor. In addition, farmers resort to selling raw materials directly, resulting in less profits. Compared with farmers, medical wholesalers process and grade Cistanches Herba. However, storage of Cistanches Herba is usually an important factor that affects its quality, and also limits its circulation in the market, which is one reason for the high price. Moreover, to obtain higher profits, we found that medical wholesalers often retail *C. salsa* as a substitute for Cistanches Herba, which reduces product quality. Therefore, quality problems are common in these VCs, and the Cistanches Herba so produced can rarely enter traditional markets and therefore provides relatively low income for farmers.

VCs 6 and 7 mainly supply the food products market. They are mainly local individual retailers that sell online and offline. These individual retailers go through simple processing and sorting to select better products. This group deals mainly in gifts that enter the market, which are generally good-quality Cistanches Herba. The prices of these are often higher than those in conventional markets due to being better quality products than those sold in traditional medicine markets. Furthermore, selling medicinal materials via e-commerce platforms greatly reduces store costs. However, there are still many gaps in the supervision process, leading to some irregular and speculative components, the origins of which remain untraceable.

With the continuous improvement in the value of Cistanches Herba products, it is gradually being recognized and accepted by consumers. Therefore, the demand for its deeply processed products in domestic and foreign markets increases yearly, bringing higher benefits and attracting a huge potential market. Furthermore, the government actively encourages local enterprises to carry out product research and development in order to enhance benefits for farmers as well as profits for enterprises, all of which increase tax revenue for the government. VCs 4 and 5 and VCs 8–10 are placed between the traditional and deep processing market models. Concerning VCs 4 and 5, processing companies purchase directly from farmers and further process the products in order to sell to consumers as TCM or health care products. In these chains, farmers still earn lower benefits, but processing companies guarantee better quality than retailers. They engage more fully in various forms of self-regulation to improve their reputation. As a result, they increase their overall input costs while improving the safety and traceability of their products.

In VC 8, the agricultural cooperative mode of “company” + “farmer” further expands the planting area and drives farmer incomes. These companies provide effective technology and equipment that help improve planting efficiency and yield. At the same time, these companies conduct further processing research, thus improving the additional value of Cistanches Herba products and expanding the market for product demand, which in turn increases the sales of Cistanches Herba related products in multiple ways. These innovative deep processing technologies improve quality, enabling companies to obtain higher profits. The cooperative order mode guarantees the farmers a production income and reduces their market risk, which effectively stimulates the enthusiasm of local farmers to plant. In VCs 9 and 10, companies concentrate on deep processing to seek a bigger market and higher profits. These related companies employ a variety of processing methods, which allow flexibility for adjusting the variety and yield of production according to market demand and product revenues. In addition, resources are relatively adequate and the product quality is good, thereby enhancing market product space. Advanced industrial facilities and products are the key to the development of the company due to the need for better deep processing technology.

According to our survey, higher standards are being placed on the quality of materials, resulting in higher input costs. Therefore, the behavior and benefits of stakeholders, as well as the quality of the products and target markets, are all closely related. As a result, the price and quality of Cistanches Herba is higher in the gift (300–1200 CNY/kg) and deep processing markets than in traditional markets (150–800 CNY/kg). In these three major markets, the majority of sales are in the form of primary processed gifts. Compared with the price advantage of *C. tinctoria*, *C. deserticola* rarely enters the traditional medicine market, and its circulation is restricted. In conclusion, with the entry of the Cistanches Herba into the health food market, further processing and development of Cistanches Herba is expected to increase in the future.

## 4 Conclusion

*C. deserticola* is a parasitic plant growing in a unique environment, and Alxa appears to be the area that is most suitable for its growth. Regarding Cistanches Herba value chains, we have demonstrated the emergence and development of vertical integration, horizontal collaboration, and e-commerce at various levels. Vertical integration is induced by the expansion of stakeholders. VC 3 and VC 4 are the result of the vertical integration of wholesalers and processing companies via the development of downstream businesses. Among the 10 VCs, VCs 8–10 are fully vertically integrated. Farmers gain access to markets, as well as to technological and financial support by joining these vertically integrated VCs. Additionally, partial vertical integration is attributed to the reliable traceability of the products. Horizontal collaborations (e.g., agricultural cooperatives) demonstrate a higher level of communication and common goals, and bring higher incomes to relevant stakeholders. E-commerce offers a convenient and inexpensive approach for retailers to build direct access to consumers. However, quality assurance in such chains is poorly understood, and there is a need for certification of good practices in e-commerce.

On the one hand, due to the specific factors associated with Cistanches Herba cultivation, independent farmers did not have access to the latest technology and were not able to develop large-scale cultivations. Some key issues related to processing procedures as well as wholesale and retail activities, that were detected during the course of our interviews and quality analyses, indicated that earning higher profits via adulterated substitutes were hidden factors that reduced the value of products. These factors can affect the quality of herbal medicines, just as a value-added cooking process could improve the quality of foods (Chen et al., 2019). Thus, further regulation of the market may be necessary in order to eliminate adulteration by pharmaceutical wholesalers and to realize higher profits.

On the other hand, with the state supporting the development of the Cistanches Herba industry, it is predicted that planting Cistanches Herba may increase yearly in the future. However, if deep processing and terminal markets of Cistanches Herba do not develop sufficiently within the next few years, raw material obtained from Cistanches Herba will face a market environment of oversupply, causing the prices to drop. Large-scale stakeholders, such as processing-acquisition-trading integrated companies, are more capable of controlling the development of Cistanches Herba. They are supported by technology and management structures. Thus, they have an advantage in ensuring quality and stability due to being associated with deep processing and quality control. In addition, the development of agricultural cooperation promotes the standardization and scale of the Cistanches Herba planting industry, allowing the formation of a complete industrial chain. Local farmers and herdsmen have a remarkable effect on poverty alleviation and play an extremely important role in economic and social development.

Here, we have demonstrated that the quality of Cistanches Herba is affected by multiple stages of the production process, which suggests that deciding on an appropriate VC is the core of quality control. Furthermore, we demonstrated that product quality, stakeholder behavior, and revenue are interrelated. Therefore, coordination of relationships in VCs may be a strategy that may be utilized in the quality control of Cistanches Herba. Diverse VCs are induced by vertical integrations and horizontal collaborations, and this enhances both the quality of Cistanches Herba and the returns to stakeholders. Therefore, well-developed VCs may produce products with good traceability as well as quality and ensure adequate financial revenues to relevant stakeholders.

## Data Availability

The original contributions presented in the study are included in the article/Supplementary Material, further inquiries can be directed to the corresponding authors.
