# Supplementary material for: The Quality Monitoring of Cistanches Herba (Cistanche deserticola Ma): A Value Chain Perspective
Source: Front Pharmacol. 2021 Nov 5;12:782962. doi: 10.3389/fphar.2021.782962 (PMC8602053; doi:10.3389/fphar.2021.782962)
Supplement: Supplementary file 2 [file Table1.doc]

Supplementary Material

# Supplementary Figures and Tables

## Supplementary Tables

Table S1 The results of the content analyses performed on of Cistanches Herba samples

| **No.** | **Sample Collection Area** | **Echinacoside (%)** | **Acteoside (%)** | **Galactitol (%)** |
| --- | --- | --- | --- | --- |
| sample_E_1 | Ejin Banner | 0.31 | 0.93 | 6.66 |
| sample_E_2 | Ejin Banner | 0.77 | 0.80 | 3.77 |
| sample_E_3 | Ejin Banner | 0.74 | 0.22 | 4.88 |
| sample_E_4 | Ejin Banner | 0.83 | 0.31 | 10.26 |
| sample_E_5 | Ejin Banner | 0.16 | 0.66 | 7.23 |
| sample_E_6 | Ejin Banner | 1.24 | 0.19 | 4.40 |
| sample_E_7 | Ejin Banner | 0.24 | 0.53 | 2.13 |
| sample_E_8 | Ejin Banner | 0.42 | 1.67 | 5.28 |
| sample_E_9 | Ejin Banner | 0.39 | 0.35 | 7.78 |
| sample_E_10 | Ejin Banner | 0.42 | 0.47 | 4.64 |
| sample_E_11 | Ejin Banner | 0.27 | 0.15 | 3.11 |
| sample_E_12 | Ejin Banner | 0.24 | 0.14 | 2.40 |
| sample_E_13 | Ejin Banner | 0.31 | 0.91 | 5.86 |
| sample_E_14 | Ejin Banner | 0.99 | 0.63 | 5.77 |
| sample_E_15 | Ejin Banner | 0.26 | 0.14 | 1.12 |
| sample_E_16 | Ejin Banner | 0.37 | 0.15 | 2.01 |
| sample_E_17 | Ejin Banner | 0.97 | 1.15 | 6.15 |
| sample_E_18 | Ejin Banner | 1.91 | 0.67 | 5.78 |
| sample_E_19 | Ejin Banner | 1.10 | 1.08 | 6.09 |
| sample_E_20 | Ejin Banner | 0.17 | 0.52 | 4.68 |
| sample_E_21 | Ejin Banner | 0.16 | 0.63 | 12.60 |
| sample_E_22 | Ejin Banner | 0.30 | 0.23 | 7.54 |
| sample_L_1 | Alxa Left Banner | 0.54 | 0.59 | 6.95 |
| sample_L_2 | Alxa Left Banner | 1.14 | 0.39 | 2.71 |
| sample_L_3 | Alxa Left Banner | 0.16 | 0.09 | 4.5 |
| sample_L_4 | Alxa Left Banner | 0.29 | 0.30 | 10.06 |
| sample_L_5 | Alxa Left Banner | 1.37 | 0.34 | 1.58 |
| sample_L_6 | Alxa Left Banner | 1.07 | 0.99 | 8.63 |
| sample_L_7 | Alxa Left Banner | 0.31 | 0.05 | 0.22 |
| sample_L_8 | Alxa Left Banner | 0.36 | 0.72 | 9.74 |
| sample_L_9 | Alxa Left Banner | 0.76 | 1.23 | 12.05 |
| sample_L_10 | Alxa Left Banner | 0.84 | 0.61 | 6.36 |
| sample_L_11 | Alxa Left Banner | 0.21 | 0.18 | 6.31 |
| sample_L_12 | Alxa Left Banner | 0.71 | 0.75 | 12.5 |
| sample_L_13 | Alxa Left Banner | 0.31 | 0.08 | 2.05 |
| sample_L_14 | Alxa Left Banner | 0.38 | 0.10 | 5.36 |
| sample_L_15 | Alxa Left Banner | 0.12 | 0.10 | 7.99 |
| sample_L_16 | Alxa Left Banner | 0.21 | 0.05 | 4.97 |
| sample_L_17 | Alxa Left Banner | 0.12 | 0.05 | 7.58 |
| sample_L_18 | Alxa Left Banner | 0.19 | 0.11 | 9.08 |
| sample_R_1 | Alxa Right Banner | 0.26 | 0.14 | 13.20 |
| sample_R_2 | Alxa Right Banner | 0.75 | 0.27 | 6.23 |
| sample_R_3 | Alxa Right Banner | 0.90 | 0.19 | 7.23 |
| sample_R_4 | Alxa Right Banner | 0.26 | 0.23 | 4.18 |
| sample_R_5 | Alxa Right Banner | 0.75 | 0.47 | 4.74 |
| sample_R_6 | Alxa Right Banner | 0.28 | 0.19 | 4.01 |
| sample_R_7 | Alxa Right Banner | 0.28 | 0.19 | 7.26 |
| sample_R_8 | Alxa Right Banner | 0.30 | 0.15 | 5.67 |
| sample_R_9 | Alxa Right Banner | 1.20 | 0.22 | 3.14 |
| sample_R_10 | Alxa Right Banner | 0.33 | 0.49 | 5.61 |
| sample_R_11 | Alxa Right Banner | 0.54 | 0.56 | 7.61 |
| sample_R_12 | Alxa Right Banner | 0.28 | 0.15 | 4.50 |
| sample_R_13 | Alxa Right Banner | 0.24 | 0.22 | 6.03 |
| sample_R_14 | Alxa Right Banner | 0.39 | 0.93 | 9.33 |
| sample_R_15 | Alxa Right Banner | 1.33 | 0.32 | 12.86 |
| sample_R_16 | Alxa Right Banner | 0.71 | 0.55 | 10.63 |
| sample_R_17 | Alxa Right Banner | 0.83 | 0.59 | 6.05 |
| sample_R_18 | Alxa Right Banner | 0.57 | 0.66 | 7.13 |
| sample_R_19 | Alxa Right Banner | 0.70 | 0.31 | 7.74 |
| sample_R_20 | Alxa Right Banner | 0.60 | 0.23 | 5.19 |
| sample_R_21 | Alxa Right Banner | 1.30 | 0.98 | 14.12 |
| sample_R_22 | Alxa Right Banner | 1.17 | 1.22 | 5.93 |
| sample_R_23 | Alxa Right Banner | 0.23 | 0.17 | 6.44 |
| sample_R_24 | Alxa Right Banner | 1.86 | 1.29 | 6.45 |
| sample_R_25 | Alxa Right Banner | 0.93 | 0.54 | 8.30 |
| sample_R_26 | Alxa Right Banner | 0.35 | 0.14 | 7.14 |
| sample_R_27 | Alxa Right Banner | 0.53 | 1.10 | 8.99 |
| sample_R_28 | Alxa Right Banner | 1.28 | 0.40 | 7.49 |
| sample_R_29 | Alxa Right Banner | 1.28 | 0.33 | 3.37 |
| sample_R_30 | Alxa Right Banner | 0.97 | 0.50 | 13.84 |
| sample_R_31 | Alxa Right Banner | 1.16 | 0.61 | 4.66 |
| sample_R_32 | Alxa Right Banner | 1.24 | 0.89 | 6.30 |
| sample_R_33 | Alxa Right Banner | 1.02 | 0.92 | 5.79 |
| sample_R_34 | Alxa Right Banner | 0.88 | 1.26 | 4.49 |
| sample_R_35 | Alxa Right Banner | 1.89 | 0.56 | 5.72 |
| sample_R_36 | Alxa Right Banner | 0.33 | 0.64 | 8.38 |
| sample_R_37 | Alxa Right Banner | 0.64 | 0.60 | 7.36 |
| sample_R_38 | Alxa Right Banner | 0.59 | 0.60 | 6.23 |
| sample_R_39 | Alxa Right Banner | 0.71 | 0.34 | 7.31 |
| sample_R_40 | Alxa Right Banner | 1.14 | 0.93 | 6.82 |
| sample_R_41 | Alxa Right Banner | 1.40 | 1.00 | 5.16 |
| sample_R_42 | Alxa Right Banner | 1.68 | 1.08 | 4.89 |
| sample_R_43 | Alxa Right Banner | 0.34 | 0.86 | 9.69 |
| sample_R_44 | Alxa Right Banner | 1.43 | 1.08 | 6.24 |
| sample_R_45 | Alxa Right Banner | 1.94 | 0.78 | 3.50 |
| sample_R_46 | Alxa Right Banner | 1.77 | 0.63 | 6.70 |
| sample_R_47 | Alxa Right Banner | 1.06 | 0.70 | 5.62 |
| sample_R_48 | Alxa Right Banner | 1.94 | 1.27 | 3.40 |
| sample_R_49 | Alxa Right Banner | 1.07 | 0.75 | 2.69 |
| sample_R_50 | Alxa Right Banner | 1.23 | 1.02 | 1.81 |

(sample_E: the sample of Ejin Bannerr; sample_R: the sample of Alxa Right Banner; sample_L: the sample of Alxa Left Banner).

Table S2 The results of the diameter and length analyses performed on of Cistanches Herba samples

| No. | Sample Collection Area | Length (cm) | Diameter (cm) |
| --- | --- | --- | --- |
| sample_1 | An’guo Chinese herbal medicine market | 30.2 | 4.2 |
| sample_2 | An’guo Chinese herbal medicine market | 30.1 | 4 |
| sample_3 | An’guo Chinese herbal medicine market | 26 | 3.9 |
| sample_4 | An’guo Chinese herbal medicine market | 25.2 | 3.7 |
| sample_5 | An’guo Chinese herbal medicine market | 24.5 | 3.8 |
| sample_6 | An’guo Chinese herbal medicine market | 25.6 | 2.9 |
| sample_7 | An’guo Chinese herbal medicine market | 16.6 | 2.4 |
| sample_8 | An’guo Chinese herbal medicine market | 14.7 | 2.4 |
| sample_9 | An’guo Chinese herbal medicine market | 15.8 | 2.9 |
| sample_10 | An’guo Chinese herbal medicine market | 26.8 | 4 |
| sample_11 | Bozhou Chinese herbal medicine market | 21.5 | 3.6 |
| sample_12 | Bozhou Chinese herbal medicine market | 23.5 | 3.1 |
| sample_13 | Bozhou Chinese herbal medicine market | 15.3 | 2.1 |
| sample_14 | Bozhou Chinese herbal medicine market | 16.8 | 2.3 |
| sample_15 | Bozhou Chinese herbal medicine market | 17.8 | 3 |
| sample_16 | Bozhou Chinese herbal medicine market | 25 | 3.1 |
| sample_17 | Yulin Chinese herbal medicine market | 23.6 | 3.6 |
| sample_18 | Yulin Chinese herbal medicine market | 25.8 | 3.2 |
| sample_19 | Yulin Chinese herbal medicine market | 30 | 3.8 |
| sample_20 | Yulin Chinese herbal medicine market | 15.1 | 2.3 |
| sample_21 | Yulin Chinese herbal medicine market | 15.6 | 2.1 |
| sample_22 | Yulin Chinese herbal medicine market | 15 | 2.6 |
| sample_23 | Ejin Banner purchasing stations | 31.5 | 4 |
| sample_24 | Ejin Banner purchasing stations | 30.2 | 3.7 |
| sample_25 | Ningxia purchasing stations | 14.2 | 2.8 |
| sample_26 | Ningxia purchasing stations | 15 | 2.6 |
| sample_27 | Ningxia purchasing stations | 14.3 | 2.8 |
| sample_28 | Gansu purchasing stations | 25.6 | 3.2 |
| sample_29 | Gansu purchasing stations | 27.3 | 3.6 |
| sample_30 | Alxa Left Banner purchasing stations | 31 | 4 |
| sample_31 | Alxa Left Banner purchasing stations | 32 | 3.9 |
| sample_32 | Inner Mongolia purchasing stations | 28.9 | 3.9 |
| sample_33 | Inner Mongolia purchasing stations | 26.1 | 3.7 |
| sample_34 | Alxa Right Banner purchasing stations | 34 | 4.5 |
| sample_35 | Alxa Right Banner purchasing stations | 32 | 4.1 |
| sample_36 | Xinjiang purchasing stations | 24.7 | 3.6 |
| sample_37 | Xinjiang purchasing stations | 24.7 | 3.2 |
| sample_38 | Xinjiang purchasing stations | 14.7 | 2.9 |
| sample_39 | Xinjiang purchasing stations | 22 | 3.5 |
| sample_40 | Xinjiang purchasing stations | 23.2 | 3.1 |

Table S3 The origin of the total 130 Cistanches Herba samples

| Type | Source | Number |
| --- | --- | --- |
| Field | Ejin Banner | 22 |
|  | Alxa Left Banner | 18 |
|  | Alxa Right Banner | 50 |
| Herbal medicine market | An’guo Chinese herbal medicine market | 10 |
|  | Bozhou Chinese herbal medicine market | 6 |
|  | Yulin Chinese herbal medicine market | 6 |
| Medicine purchasing stations | Ejin Banner purchasing stations | 2 |
|  | Ningxia purchasing stations | 3 |
|  | Alxa Left Banner purchasing stations | 2 |
|  | Gansu purchasing stations | 2 |
|  | Alxa Right Banner purchasing stations | 2 |
|  | Inner Mongolia purchasing stations | 2 |
|  | Xinjiang purchasing stations | 5 |
| Total | | 130 |

## Supplementary Figures


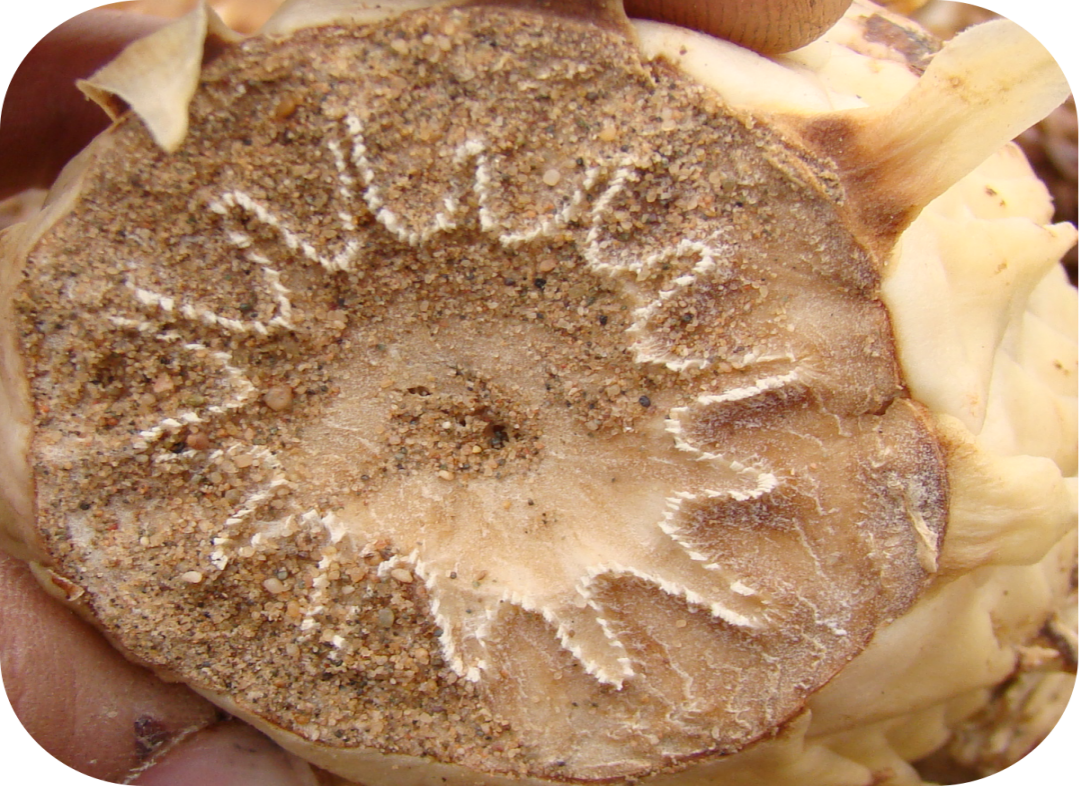


(A)


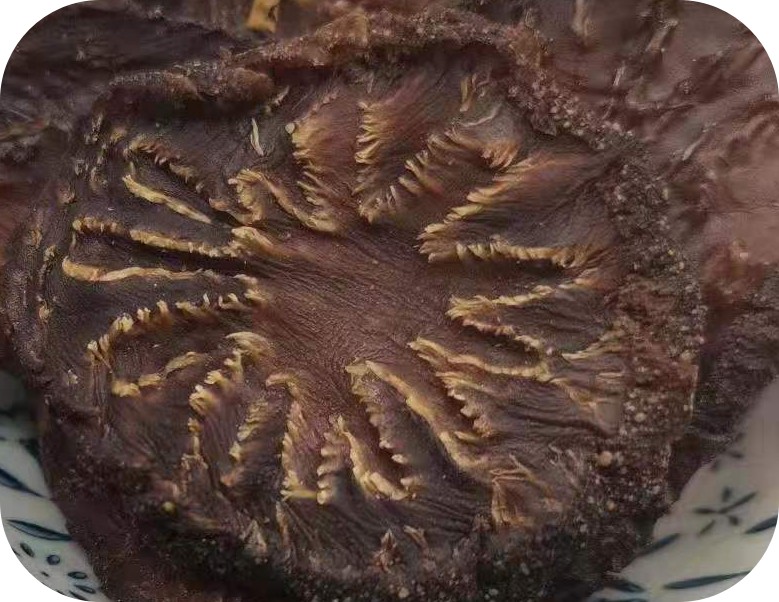


(B)


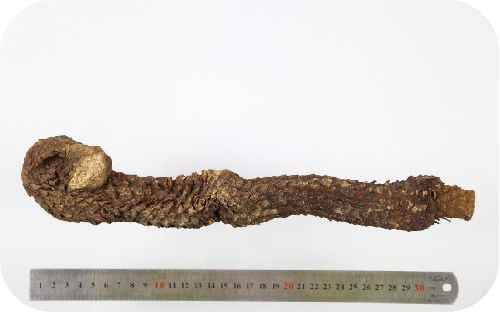


(C)

**Supplementary Figure 1.** The surface characteristics of Cistanches Herba.

(A-B: The cross section characteristic of Cistanches Herba, and B is treated by drying; C: the shape and surface characteristic of Cistanches Herba).


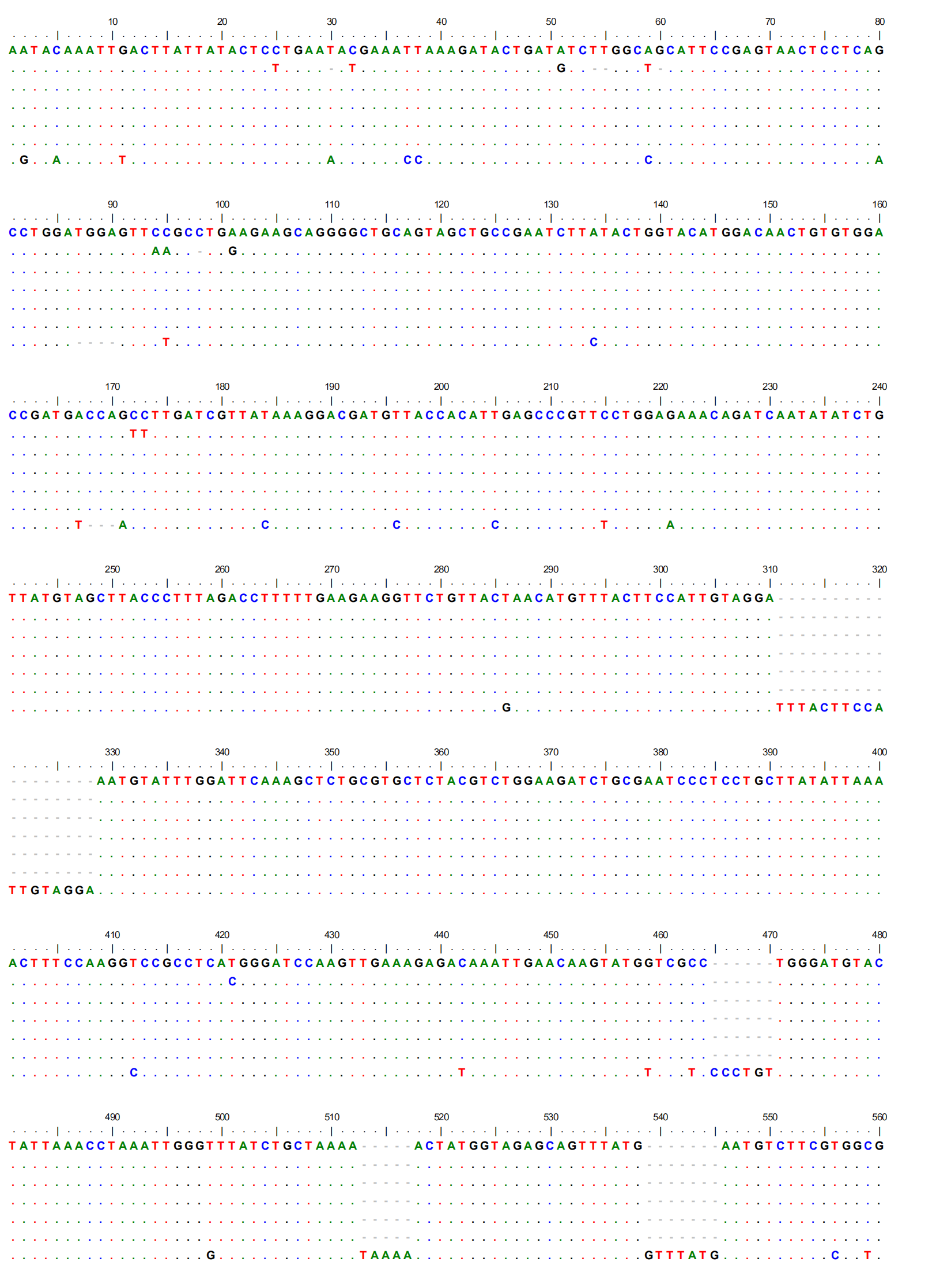


**Supplementary Figure 2.** The results of *rbc*L sequence alignment in Cistanches Herbain in Alxa region (the generally readable format is displayed in excel files).


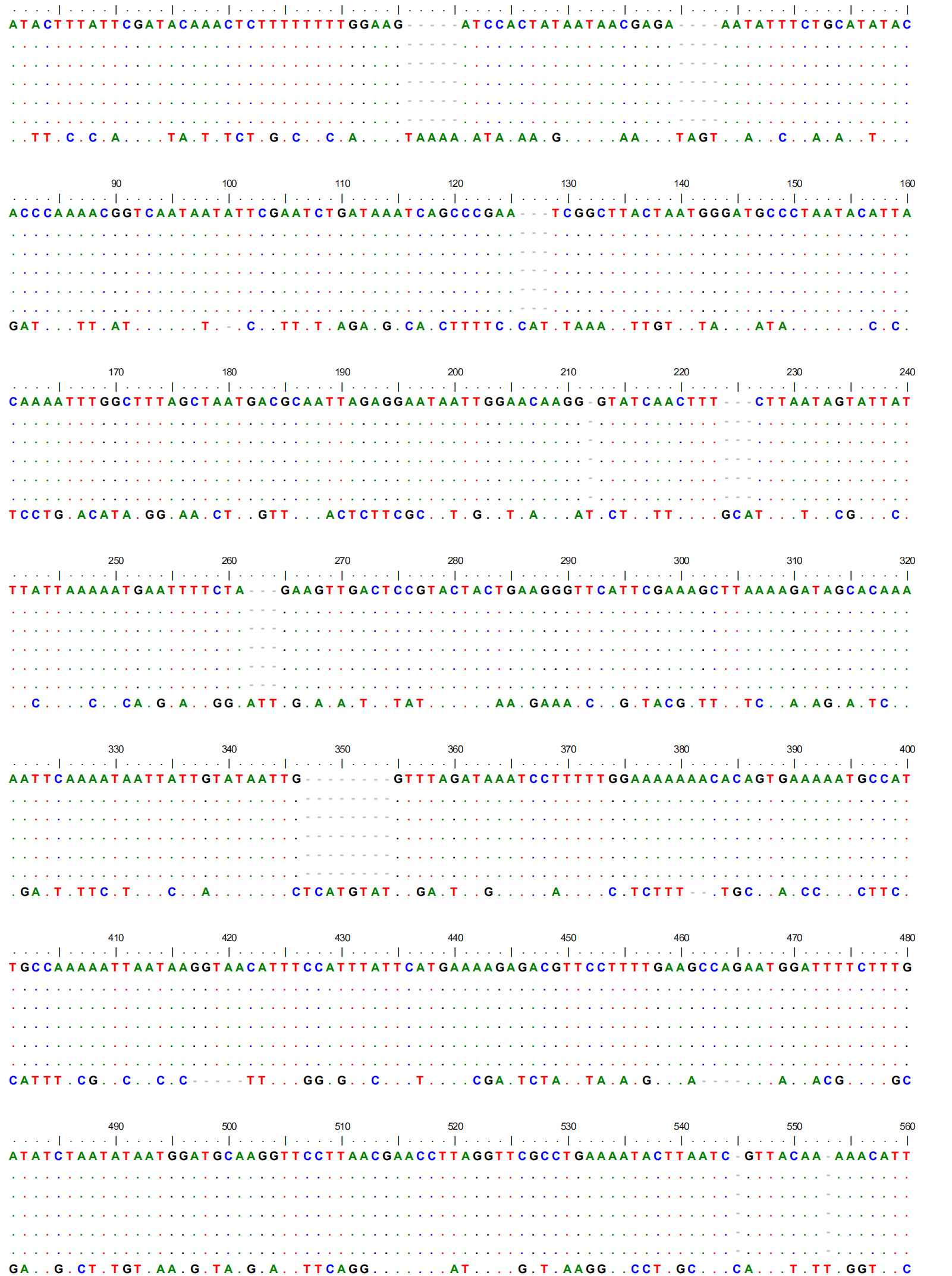

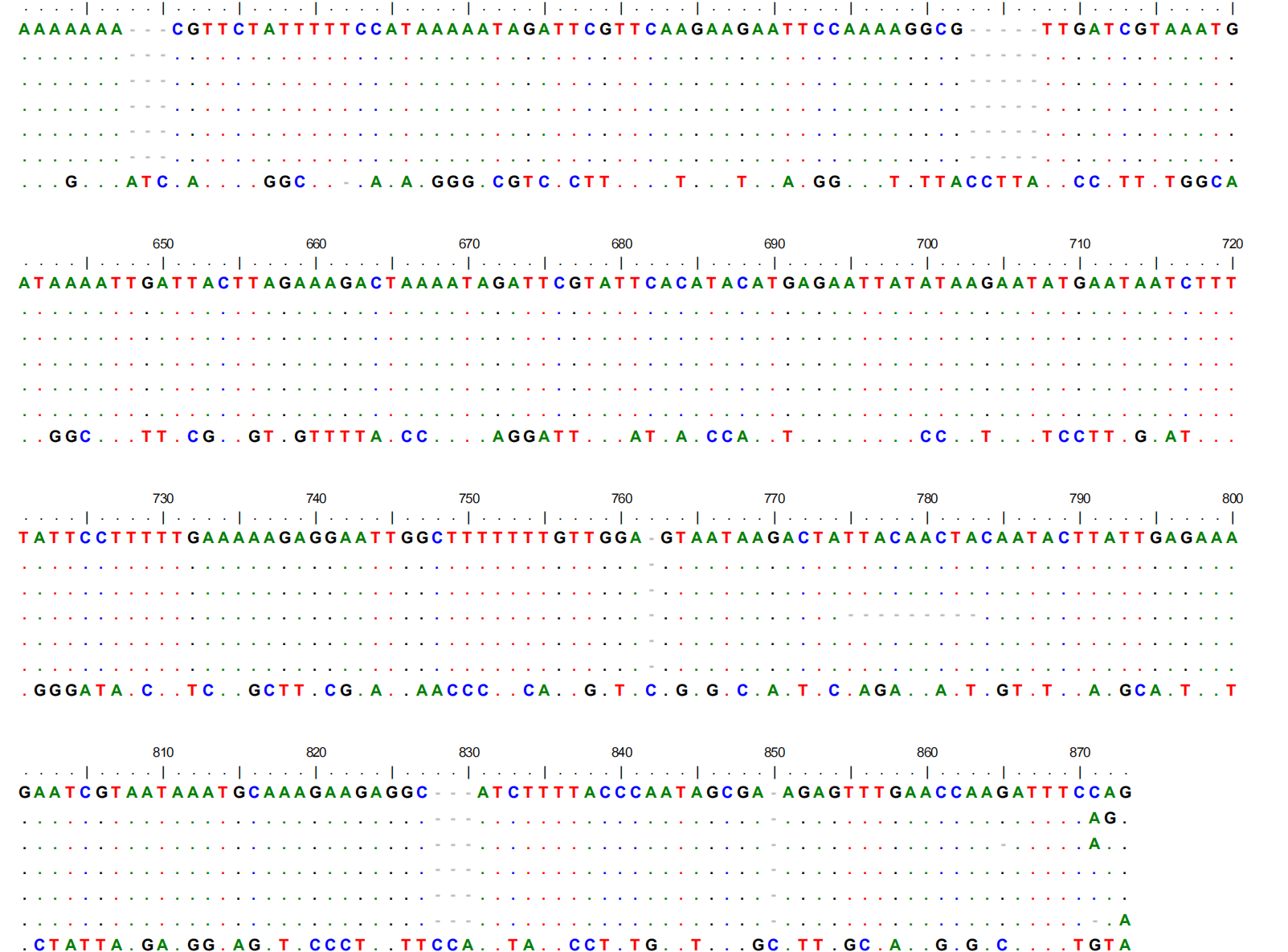


**Supplementary Figure 3.** The results of *mat*K sequence alignment in Cistanches Herbain in Alxa region (the generally readable format is displayed in excel files).


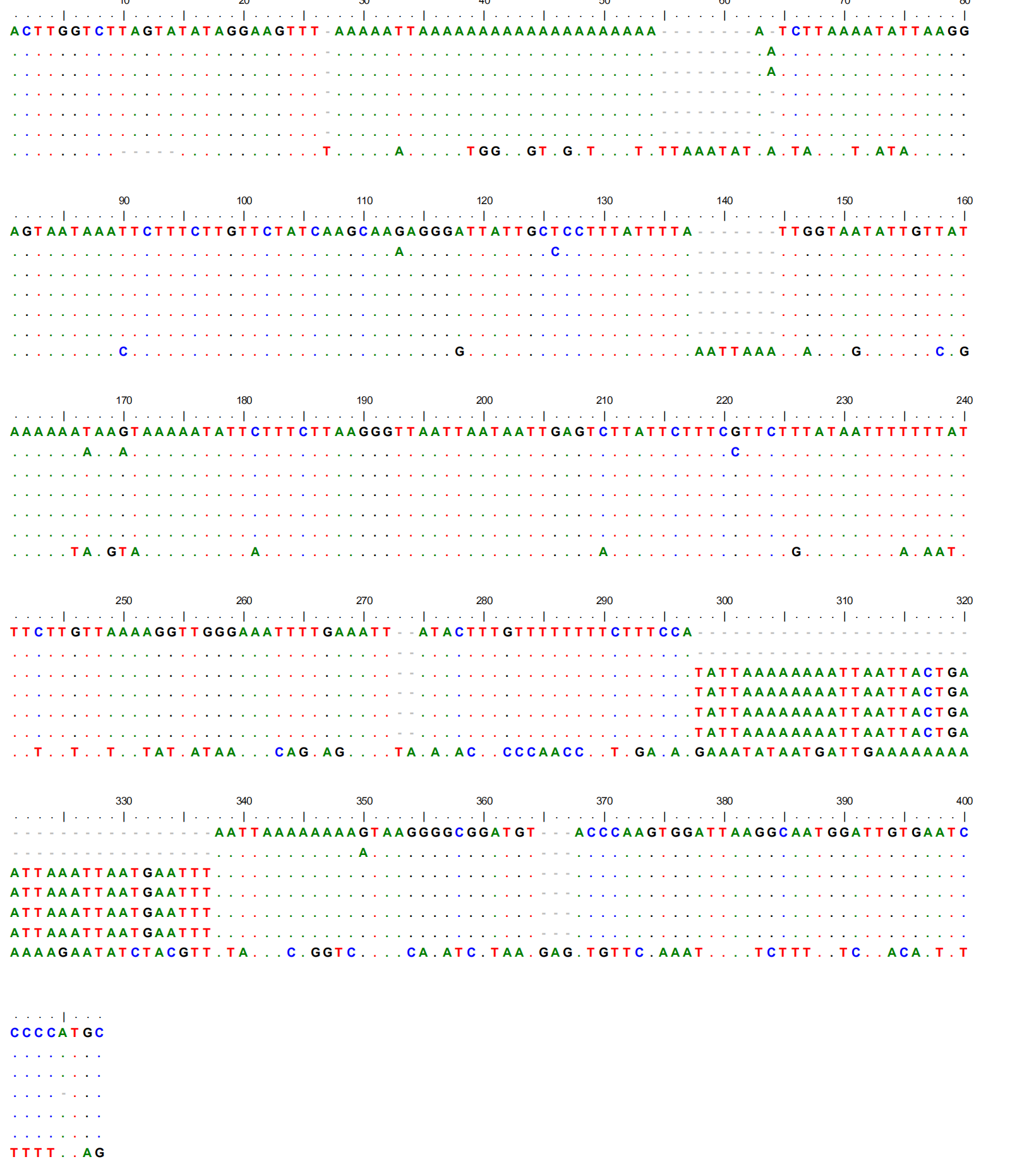


**Supplementary Figure 4.** The results of *trn*H-*psb*A sequence alignment in Cistanches Herbain in Alxa region (the generally readable format is displayed in excel files).


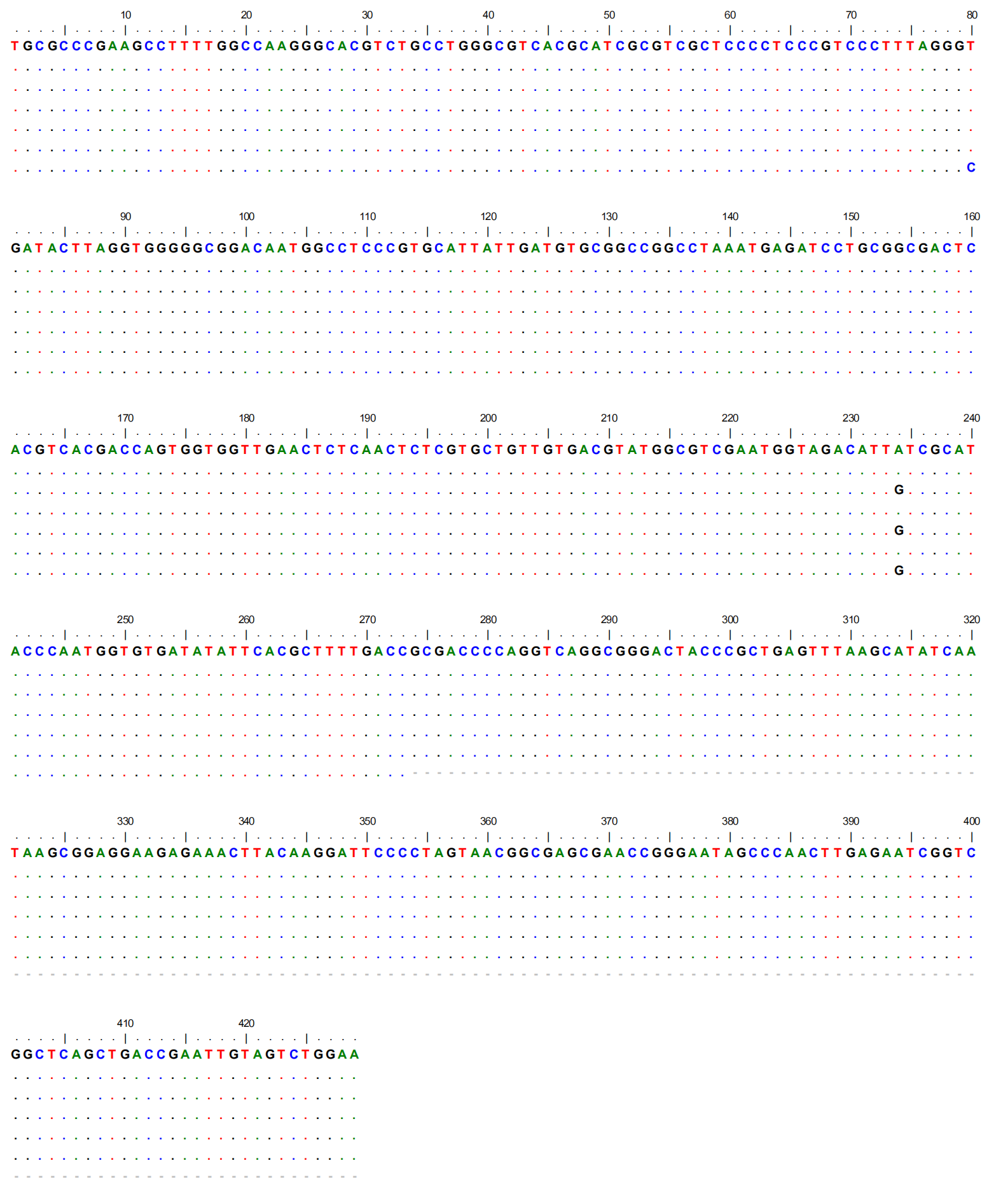


**Supplementary Figure 5.** The results of ITS sequence alignment in Cistanches Herbain in Alxa region (the generally readable format is displayed in excel files).

**Supplementary Figure 6. HPLC chromatogram of phenylethanoid glycosides in Cistanches Herba.**

Echinacoside reference standards

Acteoside reference standards

**Echinacoside**

**Acteoside**

sample_E_2

**Echinacoside**

**Acteoside**

sample_E_4

**Echinacoside**

**Acteoside**

sample_E_7

**Echinacoside**

**Acteoside**

sample_E_18

**Echinacoside**

**Acteoside**

sample_R_2

**Echinacoside**

**Acteoside**

sample_R_12

**Echinacoside**

**Acteoside**

sample_R_25

**Echinacoside**

**Acteoside**

sample_R_30

**Echinacoside**

**Acteoside**

**Echinacoside**

sample_R_31

**Acteoside**

**Echinacoside**

sample_R_36

**Supplementary Figure 7. HPLC chromatogram of galactitol in Cistanches Herba.**

Galactitol reference standards

**Galactitol**

sample_E_1

**Galactitol**

sample_E_5

**Galactitol**

sample_E_10

**Galactitol**

sample_E_18

**Galactitol**

sample_R_10

**Galactitol**

**Galactitol**

**Galactitol**

sample_R_18

**Galactitol**

sample_R_32

**Galactitol**

sample_R_33

**Galactitol**

sample_L_06

**Galactitol**

sample_L_09
